# APOBEC1 complementation factor (A1CF) is dispensable for C-to-U RNA editing in vivo

**DOI:** 10.1261/rna.058818.116

**Published:** 2017-04

**Authors:** Elizabeth M. Snyder, Christopher McCarty, Adrienne Mehalow, Karen L. Svenson, Stephen A. Murray, Ron Korstanje, Robert E. Braun

**Affiliations:** The Jackson Laboratory, Bar Harbor, Maine 04609, USA

**Keywords:** RNA editing, ACF, APOBEC1, APOB, kidney

## Abstract

Editing of the human and murine *ApoB* mRNA by APOBEC1, the catalytic enzyme of the protein complex that catalyzes C-to-U RNA editing, creates an internal stop codon within the APOB coding sequence, generating two protein isoforms. It has been long held that APOBEC1-mediated editing activity is dependent on the RNA binding protein A1CF. The function of A1CF in adult tissues has not been reported because a previously reported null allele displays embryonic lethality. This work aimed to address the function of A1CF in adult mouse tissues using a conditional *A1cf* allele. Unexpectedly, *A1cf*-null mice were viable and fertile with modest defects in hematopoietic, immune, and metabolic parameters. C-to-U RNA editing was quantified for multiple targets, including *ApoB*, in the small intestine and liver. In all cases, no changes in RNA editing efficiency were observed. Blood plasma analysis demonstrated a male-specific increase in solute concentration and increased cellularity in the glomeruli of male *A1cf*-null mice. Urine analysis showed a reduction in solute concentration, suggesting abnormal water homeostasis and possible kidney abnormalities exclusive to the male. Computational identification of kidney C-to-U editing sites from polyadenylated RNA-sequencing identified a number of editing sites exclusive to the kidney. However, molecular analysis of kidney C-to-U editing showed no changes in editing efficiency with A1CF loss. Taken together, these observations demonstrate that A1CF does not act as the APOBEC1 complementation factor in vivo under normal physiological conditions and suggests new roles for A1CF, specifically within the male adult kidney.

## INTRODUCTION

Mammalian RNA editing, the modification of a nucleotide within an intact RNA molecule, occurs in two distinct forms: adenosine to inosine (A-to-I) and cytosine to uracil (C-to-U). Both types of editing influence RNA function and its regulation. Deficiency in RNA editing can lead to substantial physiological defects including embryonic or postnatal lethality in the case of A-to-I editing (Higuchi et al. 2000; Hartner et al. 2004) and metabolic disorders in the case of C-to-U editing (Nakamuta et al. 1996). In mammals, the most common form of RNA editing is A-to-I, which is both relatively widespread throughout the body and impacts a broad range of targets. In contrast, C-to-U editing is highly tissue and target dependent. Although genetic regulation of A-to-I editing is primarily dependent on *cis*-acting factors within the target RNAs themselves, C-to-U editing appears to be regulated by a limited number of *trans*-acting factors (Gu et al. 2016).

The canonical C-to-U editing event in mammals occurs in the apolipoprotein B mRNA and results in the production of two distinct proteins, the long form (ApoB-100) and the short form (ApoB-48), encoded by the same message. The short form is a product of a single C-to-U editing event that creates a stop codon upstream of the genome encoded stop (Powell et al. 1987). In humans, this editing event is observed exclusively in the small intestine while in rodents it occurs in the small intestine and, to a lesser degree, the liver (Teng et al. 1990).

The complex responsible for *ApoB* editing requires both a catalytic and RNA binding component (Davies et al. 1989; Driscoll et al. 1989) and was initially identified based on the catalytic activity of the complex (Driscoll and Casanova 1990; Smith et al. 1991). Several years after the identification of the editing complex, the catalytic component, APOBEC1 (apolipoprotein B mRNA editing enzyme catalytic subunit 1), was cloned and shown to catalyze C-to-U editing in vitro (Teng et al. 1993). However, it was evident the full editing complex required additional components aside from APOBEC1, which did not contain the necessary RNA sequence specificity to recognize the known substrate. Substrate recognition by the editing complex was further refined by the identification of an 11-nt mooring sequence within the target mRNA which, when mutated, partially or completely abrogated APOBEC1-mediated editing (Mehta and Driscoll 1998).

Lacking the necessary sequence-specific RNA binding motif, APOBEC1 must rely on a sequence-dependent RNA binding protein to confer its target specificity. In 2000, two groups identified the APOBEC1 complementation factor (ACF, recently renamed A1CF) as the likely APOBEC1 cofactor (Lellek et al. 2000; Mehta et al. 2000). Recombinant A1CF protein was shown to complement APOBEC1 in vitro editing activity and was dependent upon the *ApoB* mooring sequences (Mehta et al. 2000). Over the course of several years, many groups demonstrated the ability of A1CF to complement APOBEC1's editing capacity in vitro, relying on purified recombinant proteins (Mehta and Driscoll 2002; Chester et al. 2004) or heterologous expression systems (Chester et al. 2003; Sowden et al. 2004; Severi and Conticello 2015).

Unlike *ApoB* editing, relevant levels of which are limited to the small intestine and liver, APOBEC1 is detectable in a much wider range of tissues (Teng et al. 1993; Nakamuta et al. 1995), suggesting APOBEC1's function may not be limited to the regulation of C-to-U editing. However, while global ablation of *Apobec1* in a mouse model resulted in a complete loss of *ApoB* editing, there were no gross impacts on fecundity or fertility (Hirano et al. 1996) and only moderate impacts on serum lipoprotein profiles (Nakamuta et al. 1996). In contrast, while A1CF and APOBEC1 have very similar expression profiles in adult tissues, there was strong evidence that A1CF may have roles outside of C-to-U editing (Sowden et al. 2002; Chester et al. 2003). This hypothesis gained even more credence when a global knockout of *A1cf* (*A1cf*^*tm1Ddsn*^) was reported to display early embryonic lethality (Blanc et al. 2005), a phenotype substantially more severe than the *Apobec1* knockout mice. Given the apparent role of A1CF in early embryogenesis, it has been difficult to assess its role in adult tissues, specifically the liver and small intestine. To test the role of *A1cf* in adult liver and small intestine RNA editing, we utilized mice carrying a conditional allele of *A1cf*.

## RESULTS

### Global loss of A1CF does not result in embryonic lethality

**FIGURE 1.**
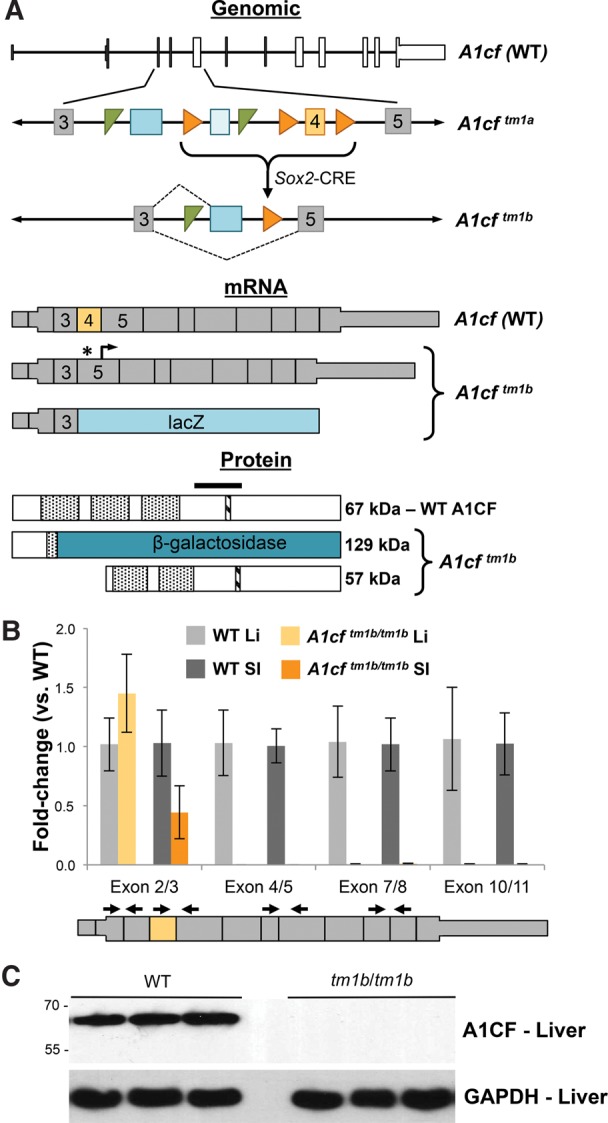
The *A1cf*^*tm1b*^ allele is a true A1CF-null allele. (*A*) Schematic of genomic, RNA, and protein impacts of the *A1cf*^*tm1b*^ allele. Genomic schematic of the WT *A1cf* allele with derivation and structure of *A1cf*^*tm1b*^. (Gray and orange boxes) *A1cf* exons; (numbers) exon position within the *A1cf* locus; (blue boxes) *lacZ* (dark) and *neo* (light) cassettes; (green triangles) FRT sites; (orange triangles) loxP sites; (dashed lines) potential splicing events from the mutant allele. The *A1cf*^*tm1b*^ allele may produce multiple mRNAs, one including the generation of a premature stop codon (*) upstream of a new 3′ open reading frame (arrow), and another encoding a chimeric transcript of *A1cf* and *lacZ*. (Thick boxes) Open reading frame. (Orange box) *tm1b* targeted exon. Schematic of WT and putative mutant A1CF proteins generated from the *A1cf*^*tm1b*^ allele. (Stippled boxes) RNA recognition motifs; (hatched box) nuclear localization signal; (black bar) antigen site for anti-A1CF antibody. (*B*) qRT-PCR analysis of the *A1cf* mRNA in WT and *tm1b* liver and small intestine. Exon junctions assessed indicated in graph and on WT mRNA schematic *below* (*n* ≥ 4, error, standard deviation). (*C*) Western blot detection of A1CF in liver with GAPDH as a representative loading control (*n* = 3 per genotype). Molecular weight in kDa indicated. (WT) Wild-type; (*tm1b*) *A1cf*^*tm1b*^.

A knockout-first conditional allele of *A1cf* (*A1cf*^*tm1a(EUCOMM)Hmgu*^) was generated by the Knockout Mouse Program (KOMP) at The Jackson Laboratory. Global *A1cf* deletion (*A1cf*^*tm1b*^) was produced by excision of the neomycin selection marker and its floxed fourth exon via *Sox2*-driven CRE-recombinase (Fig. 1A). Excision of this exon eliminates the coding sequence for the first A1CF RNA recognition motif, generates an in-frame premature stop codon, and a downstream frame-shift. This targeting strategy is designed to completely eliminate wild-type protein from the locus by generating a chimeric transcript encoding a short N-terminal peptide from the endogenous locus and full-length β-galactosidase as a reporter. However, to our surprise, heterozygous *A1cf*^*tm1b*^ crosses produced adult offspring in the expected Mendelian ratios (*n* = 57, χ^2^ = 0.579, *P*-value = 0.749) in contrast to previously reported early embryonic lethality (Blanc et al. 2005). Given the conflict between our results and the previously published report, we wondered whether the *A1cf*^*tm1b*^ allele was a true null allele. *A1cf* is highly expressed in the liver and small intestine (Dur et al. 2004), thus we tested whether homozygosity of the *A1cf*^*tm1b*^allele resulted in a loss of *A1cf* in these two tissues (Fig. 1B). qRT-PCR detection of the floxed exon and an exon boundary 5′ to the deleted exon confirmed exon 4 removal without the loss of 5′ junctions, as expected from the targeting strategy. To determine whether any mutant *A1cf* mRNA was generated from the locus, multiple exon junctions 3′ to the targeted exon were analyzed. This confirmed an almost complete loss of message in both liver and small intestine. To determine whether the mutant mRNA generated protein, Western blot analysis was used to detect A1CF in WT and mutant liver using a polyclonal antibody against the central portion of the protein. As expected, the dramatic reduction of *A1cf* message resulted in a complete loss of detectable protein (Fig. 1C). These findings demonstrate that the *A1cf*^*tm1b*^allele is a true null allele and does not result in embryonic lethality, in contrast to previous reports.

### A1CF loss does not impact C-to-U editing in the liver or small intestine

**FIGURE 2.**
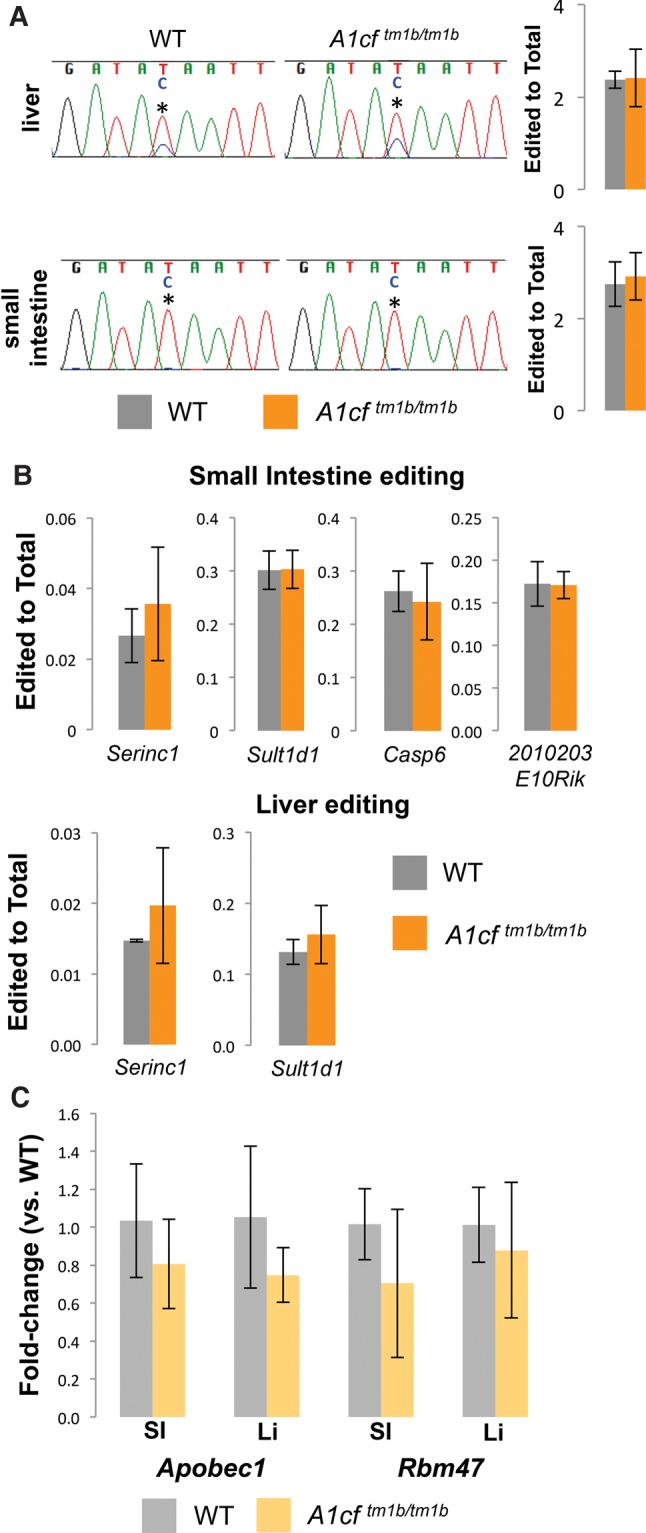
C-to-U editing is not impacted in the small intestine and liver with A1CF ablation. (*A*) Sanger sequencing of *ApoB* editing and qRT-PCR comparison of edited *ApoB* (relative to total) in wild-type and homozygous *A1cf*^*tm1b*^ small intestine and liver. (Asterisk) Editing site. (*B*) qRT-PCR comparison of multiple known editing sites in wild-type and homozygous *A1cf*^*tm1b*^ small intestine and liver. (*C*) qRT-PCR analysis of editing complex components in wild-type and homozygous *A1cf*^*tm1b*^ small intestine and liver. *n* ≥ 4, error, standard deviation. (WT) Wild-type; (*tm1b*) *A1cf*^*tm1b*^; (SI) small intestine; (Li) liver.

A1CF has long been proposed as the major cofactor for APOBEC1, the only known catalytic component of the C-to-U editing complex. APOBEC1 loss results in a complete loss of *ApoB* editing and given the viability of the *A1cf*^*tm1b/tm1b*^ mutants, we used *A1cf*^*tm1b/tm1b*^ homozygous mice to assess whether global A1CF loss altered APOBEC1-dependent C-to-U editing in the liver or small intestine, where A1CF is abundantly expressed (Sowden et al. 2004). Unexpectedly, Sanger sequencing of cDNA prepared from *A1cf*^*tm1b/tm1b*^ homozygous mutants of the canonical APOBEC1 editing target, *ApoB*, suggested no substantial change in *ApoB* C-to-U editing efficiency in either tissue (Fig. 2A). Given the nonquantitative nature of Sanger sequencing, we also utilized a previously reported qRT-PCR-based assay (Fossat et al. 2014) to quantify editing efficiency in WT and A1CF-null liver and small intestine. These analyses demonstrated no change in either editing efficiency or total *ApoB* abundance with the loss of A1CF, indicating APOBEC1 does not require A1CF to catalyze in vivo *ApoB* editing in the liver and small intestine.

Previous reports have identified a number of other APOBEC1-dependent C-to-U editing events (Rosenberg et al. 2011) that are lost with *Apobec1* mutation. Further supporting the notion that they are APOBEC1-dependent, all sites occur within stretches rich in AU sequence, similar to the known motif preference for APOBEC1 (MacGinnitie et al. 1995), and are accompanied by a downstream mooring sequence known to be required for APOBEC1 binding (Mehta and Driscoll 1998). We hypothesized that individual APOBEC1-dependent editing events may be relatively more or less sensitive to the loss of A1CF. To test whether this was the case, we compared editing efficiency across a number of previously confirmed APOBEC1-dependent RNA editing events in both the liver and the small intestine (Fig. 2B). As was the case for *ApoB* editing, we detected no difference in the editing efficiency or total mRNA abundance in any of the known APOBEC1-dependent C-to-U editing targets we examined. Based on these observations, we conclude that A1CF is not required for APOBEC1-mediated C-to-U editing in either the liver or small intestine.

Gene compensation is a commonly observed phenomenon in gene ablation models (Rossi et al. 2015). Previous work has demonstrated RBM47 to be a necessary APOBEC1 cofactor (Fossat et al. 2014) and proposed a model whereby RBM47 interacted with both APOBEC1 and A1CF to form a functional editing complex. Given this model and the knowledge that RBM47 is expressed in both the liver and the small intestine, we wondered whether loss of A1CF was being compensated for by increases in either RBM47 or APOBEC1. To test this, we assessed both *Apobec1* and *Rbm47* expression in homozygous mutant liver and small intestine (Fig. 2C). No significant changes in expression were detected for either *Apobec1* or *Rbm47* with A1CF loss, demonstrating no significant transcriptional compensation for the loss of A1CF. From this evidence, we conclude normal C-to-U editing in A1CF mutants is not the result of *Rmb47* transcriptional up-regulation.

### A1CF has novel physiological roles

As global loss of A1CF did not result in embryonic lethality, we utilized the *A1cf*^*tm1b*^ allele to determine the impact of A1CF loss on general mouse physiology. We analyzed phenotypic data generated by the JAX KOMP2 pipeline and available from the International Mouse Phenotyping Consortium (IMPC; www.mousephenotype.org/) and found significant impacts on the immune and hematopoietic system as well as altered homeostasis and metabolism. While no specific role for APOBEC1 has been reported in either the immune or hematopoietic system, knockouts do have altered cholesterol metabolism (Nakamuta et al. 1996).

Given our interest in understanding the in vivo role of A1CF as an APOBEC1 interacting factor and the known role of APOBEC1 in the mouse liver and small intestine, special attention was paid to metabolic measures associated with these tissues. Gross liver and small intestine morphology and the majority of phenotypic traits assessed showed no difference with A1CF loss. Of the analyzed traits, only the ratio of HDL to total cholesterol was significantly decreased in *A1cf*^*tm1b*^ homozygous mutants (1.0544 ± 0.0299 versus 0.9600 ± 0.0585, *P*-value = 0.0185), driven primarily by increased total cholesterol (66.6 ± 4.2 mg/dL vs. 77.6 ± 10.6 mg/dL, *P*-value = 0.0811), a finding observed only in male A1CF-null animals. This observation is in contrast to *Apobec1* knockout mice, which display decreased HDL (Nakamuta et al. 1996).

**FIGURE 3.**
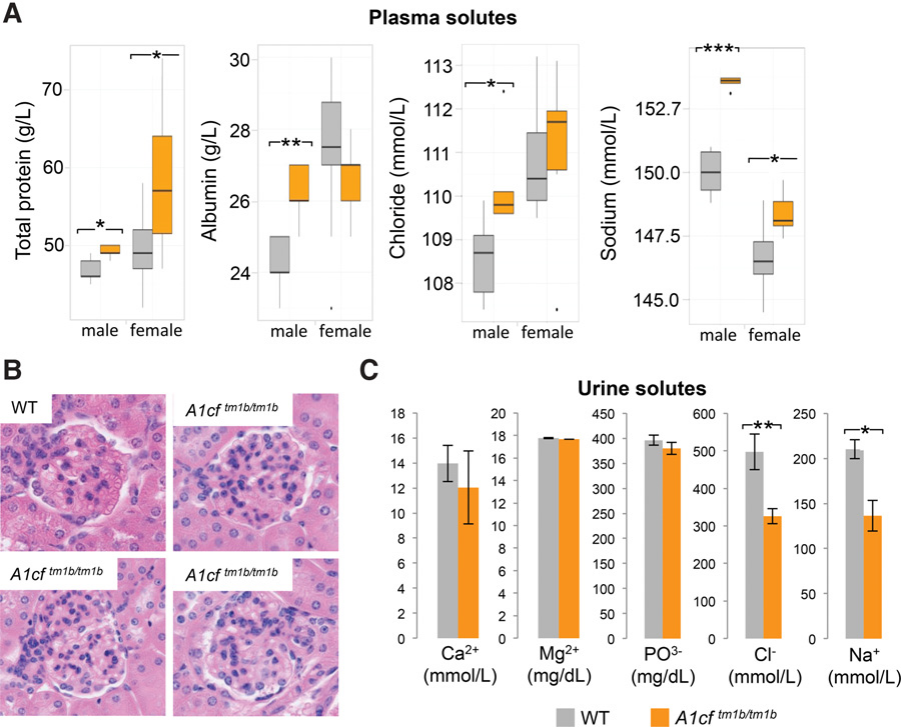
A1CF ablation impacts adult kidney physiology. (*A*) Plasma parameters in *A1cf*^*tm1b*^ homozygous animals (*n* ≥ 5 per sex per genotype combination). (Asterisks) Significant differences ([*] *P*-value <0.05, [**] *P*-value <0.01, [***] *P*-value <0.0001). (*B*) Glomerular morphology in wild-type and homozygous *A1cf*^*tm1b*^ kidney. (*C*) Urine solute concentrations in *A1cf*^*tm1b*^ homozygous animals. *n* = 3, error, standard deviation. (Ca^2+^) Calcium; (Mg^2+^) magnesium; (PO^3−^) phosphate; (Cl^−^) chloride; (Na^+^) sodium; (WT) wild-type.

A1CF has been reported by multiple groups to be highly expressed in the kidney (Lellek et al. 2000; Dur et al. 2004) and has been genetically (Pattaro et al. 2016) and molecularly (Huang et al. 2016) associated with kidney function. Given this, we evaluated whether our A1CF-null model displayed any kidney-related defects. Phenotypic analysis by the JAX KOMP2 program supported a potential role in kidney function as plasma analysis demonstrated increased protein and solute concentrations with A1CF loss (Fig. 3A), both of which indicate potential water homeostasis defects. As was the case for HDL concentrations, the impacts were more severe in males. Additionally, histological analysis of A1CF-null kidneys revealed a subtle but consistent abnormality of increased cellularity within glomeruli, again suggesting a potential water homeostasis defect in the knockout males (Fig. 3B). To examine the impact of A1CF on water homeostasis further, A1CF-null males were subjected to a multiday analysis of water intake and urine production. Over the course of the analysis, A1CF-null males showed no differences in water consumption or urine production when compared to wild-type controls. However, the concentration of both sodium and chloride was significantly decreased in A1CF-null urine as compared to wild-type (Fig. 3C). When taken in combination with the elevation of plasma solute concentration, we conclude the A1CF-null males have an underlying filtration defect, possibly due to abnormal cellularity in the kidney glomeruli.

### A1CF loss does not impact kidney RNA editing

**FIGURE 4.**
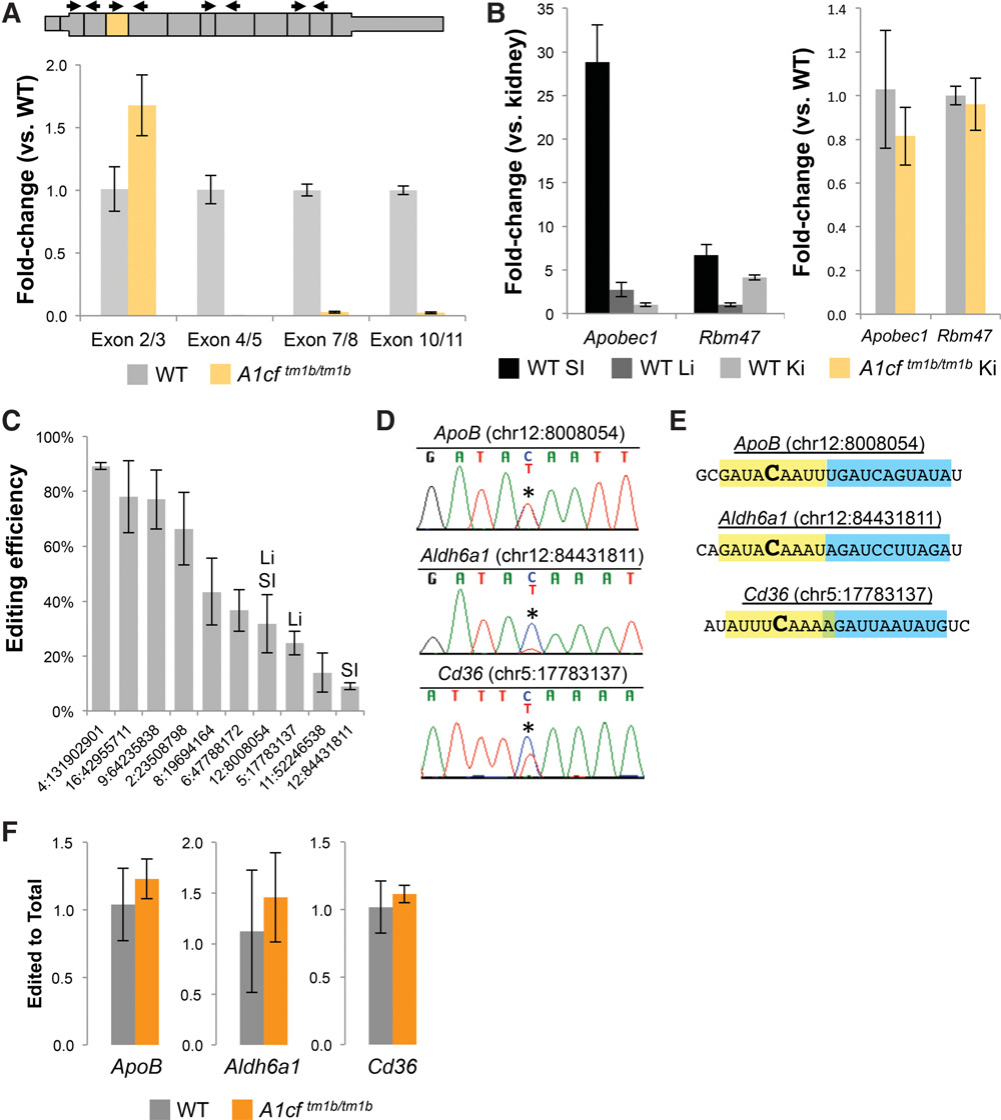
Kidney C-to-U editing occurs in a distinct set of targets and is not impacted with A1CF ablation. (*A*) *A1cf* mRNA expression in WT and *A1cf*^*tm1b*^ adult kidney assessed by qRT-PCR. Exon junctions indicated in graph and on WT mRNA schematic *above* (*n* ≥ 4, error, standard deviation). (*B*) Expression of known C-to-U editing enzymes in WT and *A1cf*^*tm1b*^ adult kidney as compared to small intestine and liver (*n* ≥ 4, error, standard deviation). (*C*) Editing efficiency at computationally defined C-to-U editing sites in the adult kidney including site overlap with known sites in the small intestine (SI) and liver (Li). *n* = 3, error, standard deviation. (*D*) Sanger sequencing confirmation of select editing sites in adult kidney. (Asterisk) Editing site. (*E*) APOBEC1 recognition site (yellow) and mooring sequence (blue) motif identification within known APOBEC1-dependent (*Apob* and *Aldh6a1*) and newly identified (*Cd36*) editing sites. (*F*) qRT-PCR comparison of edited transcript (relative to total) of select C-to-U editing sites in wild-type and homozygous *A1cf*^*tm1b*^ adult kidney *n* = 4, error, standard deviation. (WT) Wild-type.

While A1CF loss did not appear to have any impact on APOBEC1-mediated RNA editing in either the liver or the small intestine, it remained a possibility that the A1CF-null kidney phenotype was due to its function as an APOBEC1 cofactor in that tissue. Quantitative RT-PCR of *A1cf*^*tm1b*^ adult kidney confirmed a similar pattern of *A1cf* mRNA abundance as observed in both the liver and small intestine (Fig. 4A), similar to *Apobec1* and *Rbm47* expression as found in the liver, and minimal impact on *Apobec1* and *Rbm47* expression with A1CF loss (Fig. 4B). Taken together, we conclude that the impact of the *A1cf*^*tm1b*^ allele on the expression of C-to-U editing enzymes in the adult kidney is similar to what was observed in the liver and small intestine. We next asked whether C-to-U editing also occurred in the kidneys and whether it was impacted by A1CF loss. To that end, RNA sequencing of adult wild-type kidneys was examined for the presence of C-to-U editing events using a custom RNA editing identification method (Fig. 4C and Materials and Methods). From this analysis, a set of high confidence C-to-U editing sites were defined and compared to a database of RNA editing sites (darned.ucc.ie/). While the majority of sites had not been previously observed, several had been detected in the liver (Gu et al. 2012), the small intestine (Rosenberg et al. 2011), or both, suggesting that C-to-U editing of at least some targets is regulated by similar mechanisms across multiple tissues. Sanger sequencing was used to confirm kidney editing at selected sites prior to qRT-PCR analysis in the A1CF mutant kidneys (Fig. 4D). APOBEC1-mediated editing sites are characterized by an AU-rich motif around the edited site as well as a downstream motif known as the mooring sequence. Motif analysis demonstrated that *Cd36* contained both of these motifs in close association with the editing site, similar to other known APOBEC1-mediated editing events (Fig. 4E) and further validating the computational methods for C-to-U editing site identification. As was found in the small intestine and liver, A1CF loss resulted in no significant alteration of either editing efficiency or target abundance in the kidney (Fig. 4F). From these findings, we conclude that while A1CF is not required for APOBEC1-mediated C-to-U editing in vivo under normal physiological conditions, it is required for normal kidney physiology.

## DISCUSSION

To date, the consensus within the literature has held that the primary function of A1CF is as a required co-factor for APOBEC1-mediated C-to-U editing. The results reported herein demonstrate A1CF is not required for normal C-to-U editing in the case of adult liver, small intestine, or kidney. However, the observation that global A1CF loss has an impact on normal kidney physiology indicates A1CF is playing some other function, presumably nonediting related, on which the kidney is particularly reliant. This report does not represent the first suggestion that A1CF may be important in nonediting-related RNA biology. In hepatic cells, A1CF has been shown to regulate both the stability (Blanc et al. 2010) and the subcellular localization of specific mRNAs (Galloway et al. 2010). Additional work demonstrated that A1CF behaves as a nuclear shuttling protein (Blanc et al. 2003), and the shuttling is mediated by its interaction with APOBEC1 (Chester et al. 2003). These findings suggest a model whereby A1CF's primary role is to regulate the nuclear export of specific mRNA species. There is also evidence to suggest that A1CF itself is sensitive to metabolic changes (Sowden et al. 2002, 2004; Blanc et al. 2003) or disease state (Galloway et al. 2010), and it remains an open question whether altered A1CF changes affect C-to-U editing in those kinds of environments.

The lack of changes in APOBEC1-mediated editing with A1CF loss was unexpected given the abundance of in vitro evidence demonstrating A1CF interacts with and complements APOBEC1 editing activity. However, while in vitro and overexpression studies define a protein's capacity for a specific molecular function, genetic in vivo analyses are necessary to demonstrate strict reliance on that protein for a specific molecular event. Given the lack of editing changes with global A1CF loss, it appears as though while A1CF is capable of complementing APOBEC1-mediated editing in vitro, A1CF is not required for that function in vivo. This conclusion is supported by a failure to find genetic variation in C-to-U editing linked to either A1CF, or the mooring sequence for A1CF, in a genetically diverse multiparent mouse population (Gu et al. 2016). Altered editing efficiencies at the *ApoB* locus and 49 other C-to-U edited sites appeared to be solely driven by linkage to four functional *Apobec1* alleles segregating within the population. Additionally, SNPs within the mooring sequence or small indels between the edited site and the mooring sequence did not affect the editing efficiency of the RNA. It is possible other APOBEC enzymes are compensating for lack of APOBEC1 editing activity in A1CF mutants, as recent work has demonstrated human APOBEC3A is capable of catalyzing C-to-U editing in monocytes and macrophages (Sharma et al. 2015). However, this possibility seems unlikely as mouse APOBEC3 is not known to have editing activity and is only distantly related to human APOBEC3A, which has a site recognition sequence distinct from that of APOBEC1 and is not found in any of the targets examined in this work.

RBM47 has been demonstrated to be required for normal C-to-U editing in vivo as RBM47 mutants have a near complete loss of RNA editing (Fossat et al. 2014), in contrast to what was observed herein as well as in a previously reported A1CF mutant allele (Blanc et al. 2005). Additionally, APOBEC1-mediated editing in both cell and cell-free editing assays is complemented by RBM47, an activity not further enhanced by the addition of A1CF. In combination, our genetic and molecular observations in the context of A1CF loss and the observations of Fossat and coworkers suggest a model whereby RBM47 acts alone as the in vivo APOBEC1 complementation factor. Further experimental validation will be required to exhaustively test this model. It should be noted, this model does not exclude the possibility that A1CF plays some other role in modulating APOBEC1 editing activity. Although not significant, many of the editing sites examined in the context of the *A1cf*^*tm1b*^ allele showed a moderate increase in editing efficiency relative to wild-type. This finding is somewhat reminiscent of the increase in editing efficiency observed in heterozygotes carrying the previously reported *A1cf* knockout allele (*A1cf*^*tm1Ddsn*^). Unfortunately, RNA editing has not been assessed in the only other known *A1cf* ablation model (Harkins and Whitton 2016).

Direct comparison of the various *A1cf* knockout alleles is complicated by the difference in the phenotypes they generate. Failing genome-wide, side-by-side comparison, it may be difficult to determine the exact cause of the differences in phenotype between the two viable global A1CF ablation models and the *A1cf*^*tm1Ddsn*^ allele. However, multiple lines of evidence suggest the *A1cf*^*tm1Ddsn*^ allele may have additional undetected genetic aberrations. Firstly, the embryonic lethality reported for *A1cf*^*tm1Ddsn*^ occurs early in gestation, between E3.5 and E7.5, and prior to the reported onset of *A1cf* expression (Blanc et al. 2005). Secondly, a recent report demonstrated that the *A1cf*^*tm1Ddsn*^ allele displays a transmission distortion ratio (Carouge et al. 2016), which is often indicative of chromosomal rearrangements such as Robertsonian translocations (Underkoffler et al. 2005). Taken together, this evidence suggests the *A1cf*^*tm1Ddsn*^ allele phenotype may be a result of additional, unintended genetic alterations unrelated to the loss of A1CF. Given our confirmation of A1CF ablation at the DNA, mRNA, and protein level in multiple adult tissues, we are confident the *A1cf* allele reported herein genuinely represents the phenotypic impact of A1CF ablation. While it is possible that both alleles represent true A1CF global nulls, it seems more reasonable that unexpected genomic impacts or genetic interactions have led to a more extreme phenotype in the previously reported model.

## MATERIALS AND METHODS

### Production and maintenance of the *A1cf*^*tm1b*^ allele

All animal work used in this study was approved by The Jackson Laboratory Animal Care and Use Committee (Permit Number: 07007) and are in accordance with the “Guide for the Care and Use of Experimental Animals” established by the National Institutes of Health (1996, revised 2011). Animals were maintained in a 12-h light and 12-h dark cycle vivarium and provided water and autoclaved pelleted 5K52 diet (6% fat) ad libitum. Animals carrying the *A1cf*^*tm1a*^ mutation (*A1cf*^*tm1a(EUCOMM)Hmgu*^) were generated by the Jackson Lab KOMP2 program. Details of its generation and structure are provided at http://jaxmice.jax.org/strain/024325.html. *A1cf*^*tm1a*^ mice were crossed to *Sox2-Cre* carrying mice (https://www.jax.org/strain/014094) to generate *A1cf*^*tm1b*^ offspring, which were then intercrossed to produce homozygous *A1cf*^*tm1b*^ experimental animals. All experimental and control animals were maintained on a C57BL/6NJ background. Genotyping was performed using the following primers and conditions: Common_F (CCCAGGCCACCTATGAAATA) and Common_R (TCTTACCCCTCCTCGGTTTT) to generate the wild-type product and Common_F and A1cf_AF (GTTGTTAACTTGTTTATTGC) to generate the *A1cf*^*tm1b*^-specific product with the following cycling conditions: 94°C for 5 min; 94°C for 30 sec, 55.9°C for 30 sec, 72°C for 1 min (30 times); 72°C for 5 min.

### RNA isolation, reverse transcription, total template quantitative RT-PCR, and Sanger sequencing template production

RNA was isolated from wild-type and *A1cf*^*tm1b*^ small intestine, liver, and kidney using TRIzol reagent (ThermoFisher) following manufacturer recommended methods. Samples were DNase treated (RNAse free DNAse set, Qiagen) and cDNA generated using the Superscript III First-Strand Synthesis System for RT-PCR (Life Technologies) with random hexamers and following manufacturer recommended conditions. Quantitative analysis of cDNA abundance was performed using SYBR Green PCR Master Mix (Applied Biosystems) amplification on a 7500 Real Time PCR System instrument (Applied BioSystems). The following primer pairs were used for total gene abundance analysis by traditional quantitative RT-PCR: *A1cf* exon 2/3 (F—TCCAGCGCACAGGATATAGC, R—TGAAAATCTCGCAGCCCCTT), *A1cf* exon 4/5 (F—CAGGAAGCCAAGAATGCAATCA, R—TCCTCCCACAAACAATCGGC), *A1cf* exon 7/8 (F—CCGAGACTACGCTTTTGTGC, R—ATGGCTCAGAGGGTAGGTGT), *A1cf* exon 10/11 (F—TCTGCCATTGGACAAGATCA, R—GCGCTTAGCTTTGGTGGTAT), *Apobec1* (F—TACATAGCACGGCTTTATCACCAC, R—AGTCACACCGCTGCTAATAAGGTC), and *Rbm47* (F—GTCATTCCTGCGGTATCCACAC, R—CTGAACATTTGGTGCCACGG). Total gene abundance is reported as relative to the endogenous control *Rps2* (F—CTGACTCCCGACCTCTGGAAA, R—GAGCCTGGGTCCTCTGAACA). Templates for Sanger sequencing were generated using the following template-specific primer pairs: *Apob* (F—CAAGTAGCTGGTGCCAAGGA, R—TTTGTGTCCTGAGCTGCTGT), *Aldh6a1* (F—GTTGAGCCTCAAATGCAGCC, R—AGAAGCAAGCTTAAAGGCAGC), and *Cd36* (F—GGTGGTGTGTGCTCTCTCTC, R—GCTGACAGTTGCAAGCCAAA). Sequencing products were visualized using Sequencher v 5.1 (Gene Codes).

### Quantitative RT-PCR comparison of editing versus nonediting targets

SYBR Green PCR Master Mix (Applied Biosystems) amplification of total and edited templates was analyzed using a 7500 Real-Time PCR System instrument (Applied BioSystems) and total versus edited ratios compared. Primers for the following targets were as described in Fossat et al. (2014): *Apob*, *Serinc1*, *Sult1d1*, *Casp6*, and *20102302E10Rik*. Using the previously described method additional primers were designed for the following targets: *Aldh6a1* (F—GTTGAGCCTCAAATGCAGCC with either R1—GGGAGATCCTTTGATTTCTGGGT to detect total abundance, or R2—GATTTTATCTAAGGATCTATTTA to detect edited abundance) and *Cd36* (F—GGTGGTGTGTGCTCTCTCTC with either R1—GCTGACAGTTGCAAGCCAAA to detect total abundance, or R2—GTGACATATTAATCTTTTA to detect edited abundance). In all cases, relative abundance was calculated via the ddCt method with *Rps2* as an endogenous control.

### Protein isolation and immunodetection of A1CF

Total protein was isolated from small intestine, liver, and kidney by flash freezing followed by pulverization into fine powder on dry ice using a mortar and pestle. Powders were dissolved in SDS loading buffer minus dithiothreitol (100 mM Tris–Cl, pH 6.8; 4% SDS, 0.2% bromophenol blue, 20% glycerol) and boiled after the addition of 200 mM dithiothreitol followed by electrophoresis on a 10% SDS–polyacrylamide gel. Following electrophoresis, proteins were transferred to an Immobilon PVDF membrane (Millipore) and the membrane blocked for an hour in 5% nonfat dry milk in Tris-buffered saline (PBS). Primary antibodies (Atlas anti-A1CF, HPA044079, 1:500 or Abcam anti-GAPDH, 1:2500) incubation occurred overnight at 4°C, followed by washing in PBS with 0.1% Tween-20 and secondary antibody (BioRad anti-rabbit HRP, 172-1019, 1:5000) incubation. Following incubation, the membrane was washed with PBS with 0.1% Tween-20 and developed using SuperSignal West Pico Chemiluminescent Substrate (Thermo Scientific) followed by imaging using a G:Box Chemi XT4 (Syngene).

### Phenotypic analyses

Clinical plasma chemistry and body composition phenotypic data for *A1cf*^*tm1b*^ mutant mice were generated by the JAX KOMP2 Phenotyping pipeline. Phenotyping details and data are available from the International Knockout Mouse Consortium (IMPC). Wild-type and mutant data points were matched based on animal date of birth and assay date. Statistical analyses were performed using JMP 11. Tissue collection, embedding, sectioning, and hematoxylin/eosin staining for histological evaluation were performed by Histological Services at The Jackson Laboratory. Detailed histological evaluation was performed by a board certified staff pathologist on at least two adult samples of each sex for each genotype. Additional metabolic parameters (urine analysis, water and food consumption, detailed weight loss, and gain) were collected for adult (42–65 d post-partum) wild-type and *A1cf*^*tm1b*^ mice using Metabolic Cages for Single Mouse (Techniplast) over a 5-d period. Urine solute concentrations were assessed on a Beckman Coulter AU600 System by xylidyl blue (magnesium), molybdate (phosphate), arsenazo (calcium), a modified version of the Jaffe procedure (creatinine), turbidimeters (albumin), or the ISE module (sodium and chloride).

### In silico editing analysis

Total male C57BL6N/J adult kidney RNA was isolated via RNeasy Mini Column (Qiagen) purification. Sequencing libraries were constructed using the Stranded Total RNA LT with Ribo-Zero TM Gold Library Prep kit (Illumina) and paired-end 100-bp reads sequenced on an Illumina HiSeq 2500 to a minimum depth of 30 million reads per sample. Computational editing identification was based on the protocol described in Ramaswami et al. (2013). In brief, quality and duplicate filtered reads were trimmed to remove random hexamer sequence and aligned to the C57BL6N/J genome via Tophat. Variants were called using the GATK UnifiedGenotyper and filtered for minimum base and mapping quality. Output sites were further filtered to those residing within exons, represented by only a single variant type, supported by at least 10 reads, occurring in >5% and <95% of total reads, and observed in all three biological replicates. Of these, variants representing C-to-T events were reported as putative C-to-U editing sites.
